# Comparison of chilling requirements of boreal and temperate tree species in Germany and North America

**DOI:** 10.1007/s00484-026-03129-0

**Published:** 2026-01-30

**Authors:** Claudia Nanninga, Rebecca A. Montgomery, Annette Menzel, Julia Laube

**Affiliations:** 1 Department of Forest Resources, University of Minnesota, 1530 Cleveland Ave N, St. Paul, MN 55108 USA; 2 TUM School of Life Sciences, Department of Ecoclimatology, Technical University of Munich, Hans-Carl-von-Carlowitz Platz 2, 85354 Freising, Germany; 3 Department of Engineering Ecology and Landscape Development, University of Applied Sciences, Am Hofgarten 6, 85354 Freising, Germany

**Keywords:** Spring phenology, Climate change, Twigs, Experiment, Budbreak, Minnesota, Bavaria

## Abstract

Chilling is an important cue in the spring phenology of boreal and temperate tree species. It is well established that increased chilling reduces the days to bud break (DTB), but the effectiveness of different cold temperatures for chilling accumulation remains unknown for most species. Depending on this effectiveness, future warmer winters could either reduce or increase chilling accumulation for different tree species, resulting in delayed or advanced bud break. This could alter primary productivity and ecological interactions. We investigated chilling effects on DTB experimentally, using twigs of boreal and temperate tree species in Minnesota, USA. (8 species), and Bavaria, Germany (6 species). We collected twigs and applied artificial chilling in cooling chambers at three different temperatures (-7/-6.5 °C; 1.5/2°C; 4.5/4°C - USA/Germany) and with two different lengths (4/8 weeks), before placing them into forcing chambers at 21 °C/16°C, 16 h photoperiod. We additionally took twigs from the same locations on three different dates and immediately placed them into the forcing chambers. In both experiments, we observed DTB. Both experiments showed that longer chilling exposure reduced DTB and that temperatures below freezing contributed to chilling accumulation. Changing the chilling temperatures had a significant effect on DTB for 8 out of the 14 species. For most species, higher chilling temperatures more effectively reduced DTB than colder temperatures. With few exceptions, species growing in Germany required less chilling, and boreal species broke bud before temperate species. Our study confirms the need to understand species-specific chilling requirements since generalizations seem inappropriate when predicting future leaf out.

**Supplementary Information:**

The online version contains supplementary material available at 10.1007/s00484-026-03129-0.

## Introduction

The spring phenology of trees is strongly affected by temperature. While warm or forcing spring temperatures eventually cause buds to break, most temperate and boreal tree species additionally respond to mid-winter chilling, defined as the accumulation of cold temperature units within a specific range (Heide 1993). After reaching a certain chilling threshold, buds are moved out of a state of deep dormancy (endo-dormancy), during which development is halted, towards a state of shallow dormancy (eco-dormancy), during which the ability to develop under favorable conditions is re-instated (Hänninen 2016). Long-term climate scenarios project disproportionally larger increases in winter temperatures in northern latitudes (Masson-Delmotte et al., 2021), which could alter the amount of chilling received, potentially changing the onset of spring events. Because the timing of budbreak has been shown to impact net primary production, ecosystem productivity, and tree distribution (Chuine et al. 2010; Gritti et al. 2013; Harrington and Gould 2015; Morin et al. 2007), it is important to accurately model these future phenological changes (see for example Gao et al. 2024). To improve the predictive capacity of these models, it is essential to better characterize the role of chilling in the phenology of temperate and boreal trees.

Chilling should be understood in the context of the two additional major phenological cues of forcing and photoperiod. The relationship between chilling and forcing is one of counterbalances: i.e., the more chilling the buds accumulate, the less forcing they require (Heide 1993; Caffarra and Donnelly 2011; Cook et al. 2012; Fu et al. 2015a; Harrington and Gould 2015; Zhang et al. 2021; Lebedev and Shestibratov 2024). As a result, a lack of chilling during the winter months can increase the need for forcing and result in delayed budbreak (Fu et al. 2015b; Asse et al. 2018; Wang et al. 2022), erratic budbreak (Harrington and Gould 2015; Morin et al. 2009), or even prevent budbreak entirely (Polgar et al. 2014). A lack of chilling can be compensated by high amounts of forcing (Bigler and Vitasse 2019; Caffarra and Donnelly 2011; Flynn and Wolkovich 2018; Heide 1993), but temperatures in the forcing range can also negate previously accumulated chilling (Rose and Cameron 2009). Because of this dual effect of warming(Morin et al. 2009) in the winter and spring, the trend towards earlier or later budbreak with climate change is generally not linear (Chuine et al. 2016; Flynn and Wolkovich 2018; Wolkovich et al. 2022). The third major cue, photoperiod, acts as a buffer to reduce the risks of early budbreak (Flynn and Wolkovich 2018; Meng et al. 2021; Wenden et al. 2020; Zohner et al. 2020), but in some species also reduces the need for forcing (Flynn and Wolkovich 2018; Fu et al. 2019a, b).

Although it is well established that increased chilling reduces the need for forcing DTB, there are still gaps in our knowledge regarding the effectiveness of different cold temperatures, i.e., temperatures suitable for chilling accumulation. This is partly due to considerable variation among species, both in the sense of the effective temperature ranges and the quantitative thresholds for effective chilling (Harrington and Gould 2015; Nanninga et al. 2017). Studies show that species can have mild, moderate, and strong needs for chilling (Nanninga et al. 2017; Polgar et al. 2014; Wenden et al. 2020), but the optimal species-specific chilling temperature ranges are not known for most species (Ettinger et al. 2020; Wenden et al. 2020; Zhang et al. 2021). It additionally remains unclear if and how effectively temperatures below freezing (< 0 °C) contribute to chilling accumulation (Chen et al. 2019; Luedeling et al. 2013; Wenden et al. 2020). For example, Man et al. (2017) found that temperature below − 3.4 °C had no chilling effect for boreal species in Ontario, and other studies suggest that below freezing temperatures do not contribute to chilling accumulation at all (Polgar and Primack 2011). This is contrary to a large body of literature that found < 0 °C temperatures to be suitable for chilling accumulation (Baumgarten et al. 2021; Ford et al. 2016; Güsewell et al. 2017; Meng et al. 2021; Nanninga et al. 2017; Zohner et al. 2020), but whether very cold or above freezing temperatures are more effective (for example see Harrington et al. 2010) remains unclear. These questions are especially relevant in very cold areas, such as one of our study sites in MN.

Chilling requirements can differ among similar species from separate locations (Misra et al. 2021; Zhang et al. 2021). High chilling requirements usually occur in species or ecotypes from warmer regions (Hänninen 1996; Myking and Heide 1995; Yang et al. 2020), such as lower altitudes (Vitasse et al. 2009; Bigler and Vitasse 2019) and latitudes (Wenden et al. 2020), or from areas with more unpredictable weather patterns. They serve a protective mechanism against late spring frost damage (Zohner et al. 2017). For example, Zohner et al. (2017) showed that temperature variability was higher in North America than in Europe, resulting in higher chilling requirements in North American species. Two recent studies from Minnesota and Ontario found that boreal species require fewer days of chilling than temperate species, i.e. they are less sensitive to changes in chilling during the winter (Man et al., 2021a; Montgomery et al. 2020). As a result, climate change could differently affect the capacity of boreal and temperate species fulfilling their chilling requirements, potentially leading to phenological asynchrony and changes in competition (Montgomery et al. 2020).

While many studies show that warmer springs have advanced the timing of budbreak around the world (Polgar et al. 2014; Yu et al. 2017; Menzel et al. 2020), others found that this trend has slowed down in the past few decades (Beil et al. 2021), possibly due to a lack of winter chilling caused by climate change (Fu et al. 2015b; Chen et al. 2019; Menzel et al. 2020; Wenden et al. 2020; Zohner et al. 2020). Whether or not a lack of chilling is indeed responsible for this trend depends on local winter temperatures and the effectiveness of these temperatures for chilling accumulation (Wang et al. 2022). Recent studies suggest that factors other than chilling might be contributing. For example, (Walde et al. 2025) demonstrated a reduction in temperature sensitivity with higher temperatures, and Menzel et al. (2020) attributed the slowing trend of advancing budbreak to a slowing increase in spring temperatures. Some species can also be categorized as ‘spring-only responders’, i.e., they only react to forcing in the spring and are insensitive to chilling (Cook et al. 2012; Pope et al. 2013). In agreement with this, phenological models sometimes perform better when only forcing is considered (Dantec et al. 2014; Fu et al. 2012). However, other recent work shows the importance of chilling in budbreak timing (Chuine et al. 2016; Hu et al. 2022; Zohner et al. 2017).

Understanding all facets of chilling and its impact on forcing requirements can help predict future budbreak by improving phenological models to inform management decisions in forestry, for urban green infrastructure (Donnelly et al. 2024), or species selection for assisted migration (Silvestro et al. 2019). Due to the interrelation of forcing and chilling, and their joint dependency on rather unknown temperature values, observational studies are limited. A need for more chilling research in the form of experiments has been expressed recently (Ettinger et al. 2020; Hänninen et al. 2019; Primack et al. 2015; Wolkovich et al. 2022; Zohner et al. 2017). However, while chilling studies are common, and while they have shown that increased chilling reduces the need for forcing (Laube et al. 2014; McKown et al. 2018; Nanninga et al. 2017), experiments that look at the actual chilling temperature and its effect on budbreak and forcing needs are rare (but see Baumgarten et al. 2021). Additionally, few experimental studies exist that compare the impact of chilling temperatures on budbreak between similar species from different continents (Zohner et al. 2017; Walde et al. 2022). Here, we ran a twig experiment to compare the effect of chilling on budbreak for boreal and temperate species in Minnesota, USA, and Bavaria, Germany. We tested the following hypotheses: (1) Below freezing temperatures contribute to chilling accumulation, hence reduce the time to budbreak; (2) The warmer chilling temperatures in the experiment (4.5 °C USA/4°C Germany) are less effective than colder chilling temperatures in the experiment (−6.5 °C USA/−7 °C Germany) at reducing the DTB. We did not test budbreak timing at chilling temperatures above the forcing threshold of 5 °C (Bigler and Vitasse 2019; Marchin et al. 2015; Vitasse and Rebetez 2018), so this experiment does not examine the upper limit of chilling effectiveness; (3) Longer chilling duration decreases the time to budbreak. (4) Boreal species have lower chilling requirements than temperate species, both in the USA and Germany. This hypothesis was tested as boreal species are found in regions with more reliably cold winters and chilling exposure. These species therefore often have lower chilling requirements as they don’t have to rely on chilling accumulation as a protective buffer from early budbreak and potential frost damage.

## Materials and methods

We conducted a chilling and greenhouse experiment across two continents with twigs of adult boreal and temperate tree species (Table 1). Twigs in the USA were collected at the Cedar Creek Ecosystem Science Reserve (CCESR) outside of East Bethel, Minnesota (MN) (N45°24’7.29’’, W93°11’57.85). Twigs in Germany were collected at Freising, Bavaria, located close to the Technical University of Munich campus (N48° 24’ 1.38566”, E11° 43’ 4.82416”). The climate at Cedar Creek is continental, with mean temperatures of on average around − 10 °C and 21.5 °C in the coldest (January) and warmest month (July) respectively (averaged from 1988 to 2014^1^). The average annual rainfall in Cedar Creek and Freising is around 660 mm and 792 mm respectively. Even though located at a higher latitude, the climate in Germany is influenced by the North Atlantic drift, resulting in on average milder winter temperatures in Freising compared to East Bethel. Mean monthly temperatures in Freising range from − 1.1 °C in the coldest (January) and 17.9 °C in the warmest (July) months (climate station Freising-Weihenstephan of the German Meteorological Service, 1981–2010).

**Table 1:** Statistical analysis (ANOVA) of the effect of chilling time (chilltime) and chilling temperature (chilltemp) on the time to Budbreak in 6 species from Germany and 8 species from the USA (Significance levels are ***highly significant, **moderately significant, *marginally significant. None of the interactions (Chilltime x Chilltemp) were significant)

Country	SPECIES	TYPE boreal = B Temperate = T	Chilltime *p*-value	Chilltime F-value, df	Chilltemp *p*-value	Chilltemp F-value, df
USA	*Larix laricina* (LaLa)	B	< 0.001^***^	*F*_1,42_=19.98	0.11	*F*_2,42_=2.26
USA	*Populus tremuloides* (PoTr)	B	< 0.001^***^	*F*_1,49_=40.29	< 0.001^***^	*F*_2,49_=15.56
USA	*Betula papyrifera* (BePa)	B	< 0.001^***^	*F*_1,53_=59.48	< 0.001^***^	*F*_2,53_=9.15
USA	*Acer saccharinum* (AcSa)	T	< 0.001^***^	*F*_1,45_=123.66	0.081	*F*_2,45_=2.64
USA	*Acer rubrum* (AcRu)	T	< 0.001^***^	*F*_1,40_=29.32	0.75	*F*_2,40_=0.28
USA	*Populus grandidentata* (PoGr)	T	0.31	*F*_1,43_=1.02	0.87	*F*_2,43_=0.13
USA	*Quercus Macrocarpa* (QuMa)	T	< 0.001^***^	*F*_1,42_=35.52	0.0083^**^	*F*_2,42_=5.37
USA	*Quercus rubra* (*QuRu*)	T	0.0011^**^	*F*_1,41_=12.15	0.018^*^	*F*_2,41_=4.40
GER	*Larix laricina* (LaLa)	B	< 0.001^***^	*F*_1,45_=13.07	0.28	*F*_2,45_=1.28
GER	*Populus tremula* (PoTre)	B	< 0.001^***^	*F*_1,53_=32.57	< 0.001^***^	*F*_2,53_=16.21
GER	*Betula pendula* (BePe)	B	0.18	*F*_1,52_=1.81	0.0039^**^	*F*_2,52_=6.16
GER	*Acer pseudoplatanus* (AcPs)	T	< 0.001^***^	*F*_1,48_=33.42	0.0019^**^	*F*_2,48_=7.13
GER	*Quercus robur* (QuRo)	T	0.15	*F*_1,46_=2.08	0.42	*F*_2,46_=0.87
GER	*Quercus rubra* (QuRu)	T	< 0.001^***^	*F*_1,46_=16.10	0.0073^**^	*F*_2,46_=5.47

**Fig. 1:**
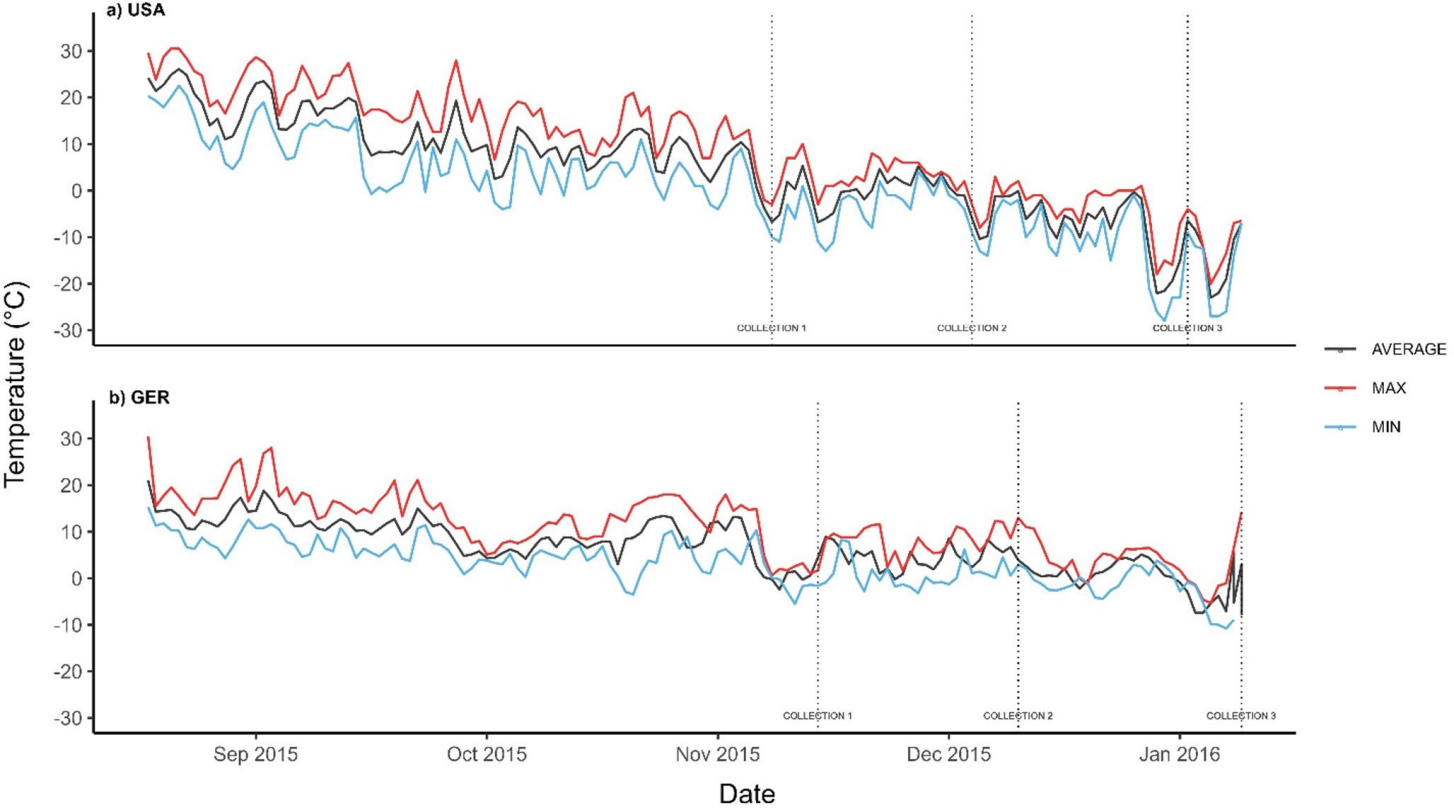
Daily ambient temperatures (average, maximum and minimum °C) in Bavaria and Minnesota starting September 1, 2015 throughout the 8 weeks of twig collection

We harvested twigs from 6 to 8 temperate and boreal tree species from Bavaria and Minnesota respectively during three collection dates from beginning of November to mid-January in winter 2015/2016. Two species were sampled in both Minnesota and Bavaria (*Larix laricina*, *Quercus rubra*), all other samples were taken of species from the same genus (e.g. Birch - USA: *Betula papyrifera*, Bavaria: *Betula pendula*). We chose 10 donor trees per species in both locations from which to collect twigs. Some samples were lost while processing: Four twigs from the *Q. rubra* collection in Bavaria, and ten twigs of *P. grandidentata*, USA, for the 4 weeks collection. The ambient temperature variations between September 1, 2015, and the end of twig collection were more extreme in Minnesota compared to Bavaria, ranging from a daily mean temperature of 13.3 °C (11/4/2015) to −23 °C (1/17/2016), compared to from 13.4 °C (11/7/2015) to −7.4 °C (1/15/2016) in Bavaria (Fig. 1).

### Experiment 1: artificial chilling in chilling chambers

To determine the effect of chilling temperatures and chilling length on timing of spring budbreak, we conducted an experiment that included a combination of chilling temperature (3 levels) and time (2 levels), i.e., six combinations (USA and Germany respectively): ~−6.5/−7 °C, ~ 1.5/2°C, ~ 4.5/4°C for a period of 4 or 8 weeks. Because chamber controls only allowed for coarse adjustment and were measured in Fahrenheit (USA) and Celsius (Germany), the temperatures in the chilling chambers varied slightly between Germany and the USA. Chilling treatments were chosen for three reasons. First, they cover a broad range of chilling (11 °C); second, temperatures far below freezing were included; and third, at chilling temperatures above the forcing threshold of 5 °C (Bigler and Vitasse 2019; Marchin et al. 2015; Vitasse and Rebetez 2018), it becomes difficult to disentangle the effect of chilling and forcing in these kind of twig experiments. This is due to the possibility that chilling and forcing are accumulated simultaneously, unless twigs are in a state of deep dormancy where forcing has no effect. However, in experiment 2 (see below), twigs from the same collection were forced immediately after collection and had a very high budbreak percentage (Appendix S2c, d), which shows that chilling requirements had been met and forcing was effective. To exclude the effect of photoperiod, twigs were chilled in the dark (24/7) following similar experiments (Sønsteby and Heide 2014; Wang et al. 2024). On November 21/27, 2015 in Minnesota and Bavaria respectively, we harvested *n* = 10 twigs per species for each of six experimental conditions for a total of *n* = 480 in MN (8 species) and *n* = 360 in Bavaria (6 species). We used only twigs with a minimum of six live buds and made sure that they had a green tissue ring at the cut end, indicating they were alive and had the potential to break bud. After cutting, the twigs were placed in iced coolers and immediately transported to the University of Minnesota St Paul campus (USA) and Technical University of Munich-Freising campus (Germany).

Twigs in the warmer two treatments were placed in small glass jars with water, and twigs in the below freezing treatment were wrapped in moist tissue inside of Ziploc bags. Glass jars were checked for water on a regular basis and water was added when evaporation had occurred. We also cut the end of the twigs and exchanged the water on a weekly basis to minimize xylem clogging and potential problems with soiled water. The twigs in the below freezing treatment were not watered or cut during the chilling period. After 4 or 8 weeks of chilling, the twigs from all treatments were immediately moved to growth chambers that were set to resemble spring-like temperatures: 21 °C day/16 °C night, 16 h photoperiod.

In the growth chambers, the twigs were scored for budbreak every 2–3 days. Budbreak was defined as at least 1 bud broken according to the Biologische Bundesanstalt, Bundessortenamt, and Chemical Industry (BBCH) 7: beginning of bud burst – first green leaf tips just visible (Meier 1997). We also recorded the percentage of twigs that were able to break bud. Dead twigs (twigs with no green tissue ring after cutting) and twigs with broken or dead buds were recorded and removed from the experiment and counted as unable to break buds. Potential causes for mortality were damage during cutting and handling, or twigs might have died during chilling treatments.

### Experiment 2: chilling at ambient temperature

To better understand the effect of chilling length and below freezing temperatures in ambient conditions on DTB, we conducted a second experiment with the same species from both countries. This experiment was especially relevant for twigs in MN, where the ambient average daily temperatures were below freezing for the entire duration of the experiment. We collected twigs from *n* = 5 donor trees during three collections (USA/Germany: November 21/27, 2015, December 17/23, 2015, January 14/22, 2016) for a total of *n* = 120 in MN (8 species) and *n* = 90 in Bavaria (6 species). Twig collection and transportation were repeated as in the chilling chamber experiment. After arrival at the universities, the twigs were immediately placed in small water jars and flushed in the warming chambers at 21 °C/16°C, 16 h photoperiod. Twigs for *P. grandidentata* at ambient temperatures after 4 weeks are missing.

Because we could not measure when exactly the trees became dormant prior to the experiment, we started collections when all leaves of the donor trees had visibly senesced. Because it is possible that chilling had already accumulated in the buds before the collection, we calculated chilling hours at ambient temperature before the collection, starting November 1. We chose an upper chilling threshold of 5 °C according to Murray et al. (1989). We chose November 1 as a random date for the initiation of chilling accumulation, because it is not uncommon for temperatures above 5 °C to occur in October in both locations, and choosing November 1 increased the likelihood of buds being dormant before the initiation of the experiment. Because the average daily temperatures in MN between December 17, 2015 and January 16, 2016 were below freezing, this experiment also allowed us to better understand the effect of below freezing temperatures on the timing of budbreak. To calculate the amount of accumulated chilling hours prior to collection, we used weather data from the CCESR (USA) and the Technical University Munich (Germany). Chilling was calculated as hours below the threshold of < 5 °C.

### Statistical analysis

All data were analyzed, and all graphs were produced with R statistical software R 3.3.2 (Crawley 2007). We examined the effect of chilling temperature, and chilling length on budbreak timing and budbreak percentage for all species in the experiment from Germany and the USA. Analytical assumptions of normality and homoscedasticity for the models were examined by graphical inspection of residuals. The effects of chilling temperature, chilling time, species, and their interaction on days to budbreak were analyzed using linear models. We initially ran a full model that included data from both Germany and the USA. However, because we found a significant difference between the data from both countries, we analyzed all the data from the USA and Germany separately.

For experiment 1 (artificial chilling in chilling chambers), we analyzed the full models for USA and Germany data using mixed effects models. The response variable, DTB of living twigs, was log(*x*) transformed and fit as a function of chilling time (discrete) and chilling temperature (discrete) and their interaction with species as random effect. To examine the response of each species to chilling time and chilling temperature on DTB, we also ran separate analyses for each species in both countries using ANOVA.

For experiment 2 (chilling at ambient temperature), for both Germany and the USA, the impact of chilling length on the timing of budbreak was analyzed using ANCOVA. The predictor species was treated as a discrete, while the predictor chillinghours was treated as numerical since twigs were chilled in situ and exposed to ambient temperature conditions. The response variable DTB was log(*x*) transformed. The second order term chilling time^^2^ *(i.e.*,* chilling time squared* to account for potential curvature of the data) was not significant for either location, so we excluded it. We chose models with higher adjusted R^2^: Germany data was analyzed without interactions and USA data included interactions.

Ambient chilling hours were calculated as hours < 10 °C accumulated in the field since November 1 st (Baumgarten et al. 2021). Because of fixed temperature conditions in the greenhouse, accumulated days to budbreak were chosen as the response variable (as opposed to GDD’s, which in our case represent a transformation of the number of days to budbreak).

## Results

### Experiment 1: artificial chilling in chilling chambers

**Fig. 2:**
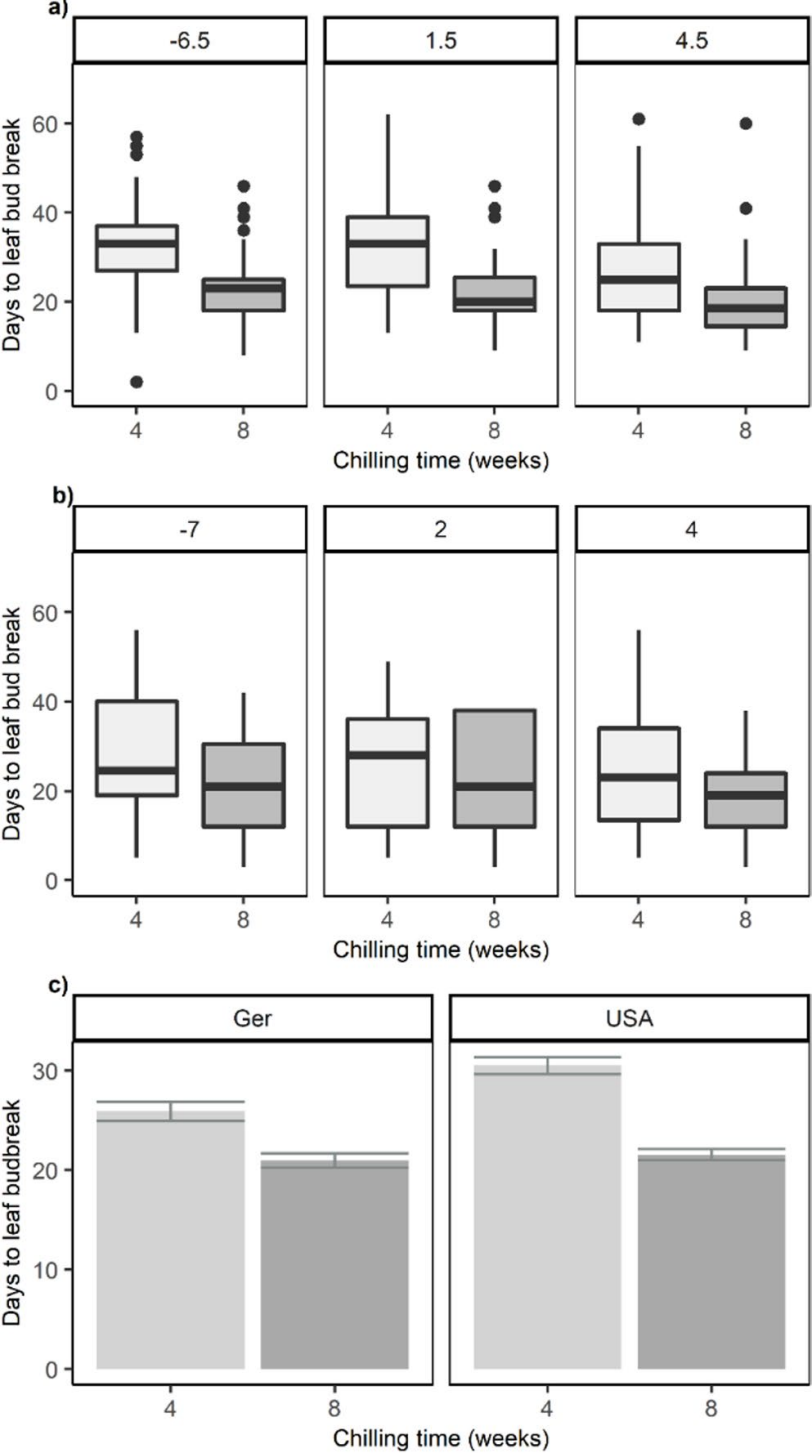
Effect of experimental chilling on the time to leaf budbreak in twigs from 8 species in the (**a**) USA and 6 species in (**b**) Germany. Twigs were chilled at 3 different temperatures and after 4 weeks (light gray) and 8 weeks (dark gray) exposed to 21°C/16°C in growth chambers until leaf budbreak. The graph shows the effect of chilling length (4 and 8 weeks) across all chilling temperatures and species in the USA and Germany. The boxplots show first quartile, median and third quartile ± minimum and maximum values within 1.5 times the interquartile range and outliers as dots. The average days required to achieve budbreak are shown in (**c**). The bars show the average of raw data and error bars show standard error

In the full models for both countries, chilling temperature and chilling time significantly affected the time to budbreak, but their interaction was not significant (USA: *log(y) = 3.67–0.08(chilling time)–0.01(chilling temperature)*; Germany: *log(y) = 3.29–0.06(chilling time)–0.01(chilling temperature*). Longer chilling time decreased the DTB (USA: *F*_1,393_=159, *P* < 0.001, Germany: *F*_1,369_=47.85, *P* < 0.001, Fig. 2), but even though chilling temperature significantly affected DTB in both countries (USA: *F*_2,392_=13.43, *P* < 0.001, Germany: *F*_2,369_=12.49, *P* < 0.001, Fig. 2), the actual temperature effect was mixed, i.e., warmer chilling could either increase or decrease DTB. Consequently, we analyzed the effect of chilling temperature and time for each species and each country separately.

**Fig. 3:**
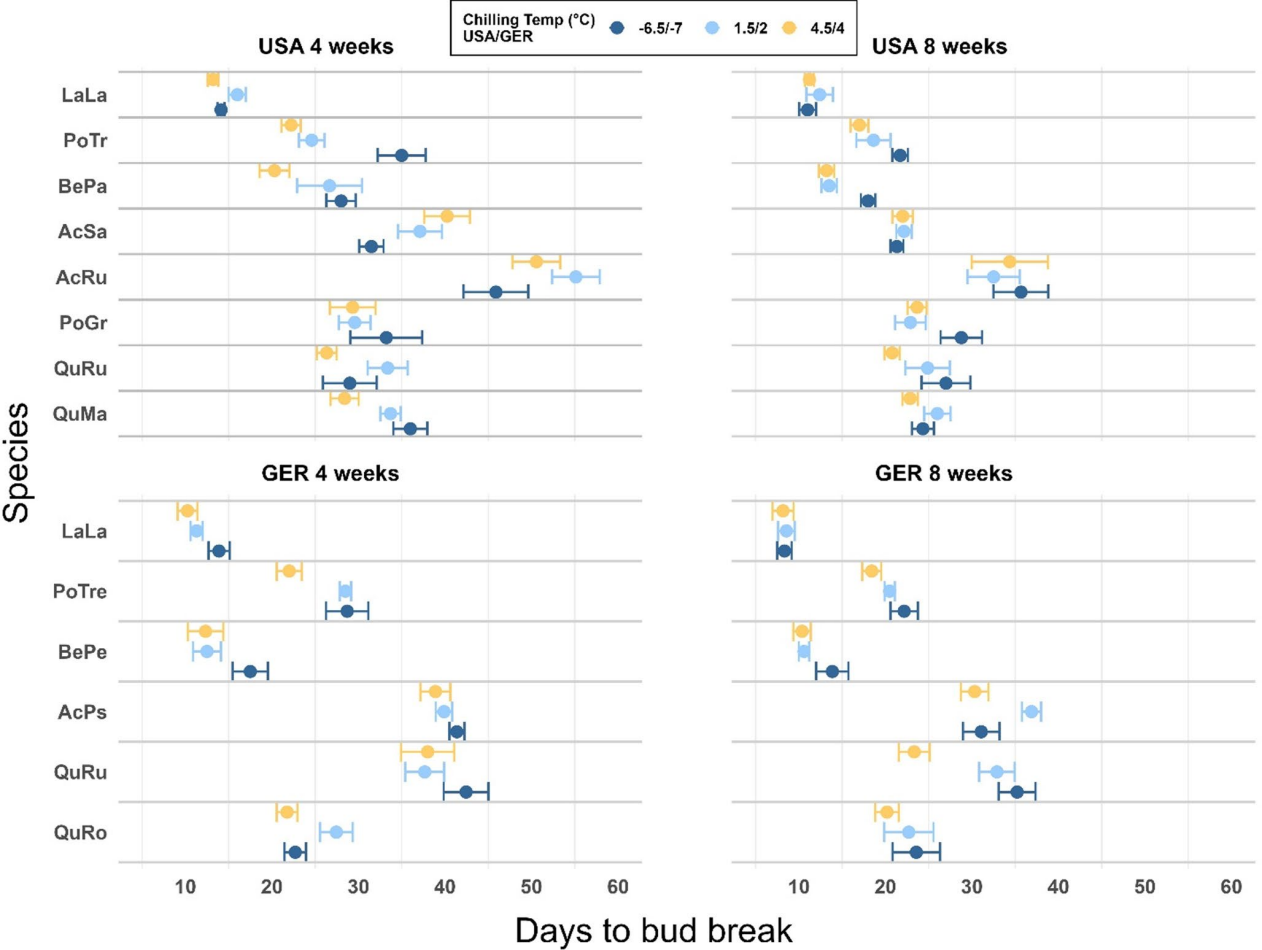
Effect of experimental chilling on the time to bud break in twigs from 8 species in the USA and 6 species in Germany. Twigs were chilled at 3 different temperatures and after 4 and 8 weeks exposed to 21°C/16°C in growth chambers until leaf buds burst. Boreal species in both the USA and Germany are listed first. The dots show the mean of the raw data, error bars are standard error

Chilling temperature significantly affected the timing to budbreak for eight out of the 14 species (Table 1; Fig. 3). All those species broke bud faster in the 1.5/2°C and 4.5/4°C treatments compared to the − 6.5/−7 °C treatment, i.e. they preferred warmer chilling temperatures (Fig. 3). However, this trend was not linear for *A. pseudoplatanus* and *Q. macrocarpa* in the 8 weeks chilling treatment. The only species in the experiment to break bud earlier at the colder chilling treatment was *A. saccharinum*, but only in the 4 weeks treatment. While not significant, this trend was very noticeable and showed that after 4 weeks of chilling, the difference in time to budbreak was around 12 days between the − 6.5 °C treatment (on average 32 ± 1 days of forcing) and the + 4.5 °C treatment (on average 40 ± 2 days of forcing), which is close to 2 weeks.

We found a significant difference in DTB among all species in the USA and Germany. In both countries, species of the *Acer* family required the longest time to break bud, while *L. laricina* required the least time (Fig. 3). For 2 of the 14 species in the experiment, DTB was neither significantly impacted by chilling time nor by temperature (*P. grandidentata*, *Q. robur*), and chilling time was not significant for *B. pendula*. For 11 out of the 14 species, chilling time had a statistically significant effect. For example, over all chilling temperatures, time to budbreak for *A. pseudoplatanus* was on average 39 (± 1) after 4 weeks, compared to 32 (± 1) after 8 weeks – a difference of close to 8 days.

When we compared the average days to budbreak for the overlapping species (*L. laricina* and *Q. rubra*) across all temperature and length treatments, we found differences between Germany and the USA (see Fig. S1 available as Supplementary Data, data not analyzed). *L. laricina* twigs from Germany broke bud on average after 10.21 (± 0.48 SE) days, while *L. laricina* twigs from the USA broke bud after an average of 12.79 (± 0.39 SE) days. This small difference of around 2.5 days was expected, given the species’ low chilling requirements. For *Q. rubra*, on the other hand, the effect was more pronounced and reversed. *Q. rubra* twigs from the USA broke bud after on average 26.61 (± 1.0 SE) days, compared to *Q. rubra* twigs from Germany, which were slower to break bud and required on average 35.65 (± 1.21 SE) days, a difference of around 9 days.

All species in the experiment, in both Germany and the USA, were able to break bud after being treated in the chilling chambers, and the budbreak percentage (survival) was generally high (on average > 60%, except for *L. laricina* in the 1.5/2°C treatment, where budbreak percentage was ~ 50%) (see Figure S2a, b available as Supplementary Data). There was no detectable pattern in the rate of survival among the three chilling treatments and between chilling time, which suggests that the chilling temperatures used in the cold chambers were within the suitable range for chilling accumulation. Additionally, after 4 and 8 weeks of ambient compared to artificial chilling, DTB in both experiments occurred very close together (see Figure S3 available as Supplementary Data), again suggesting that artificial chilling was successful in advancing budbreak.

### Experiment 2: chilling at ambient temperature

**Fig. 4:**
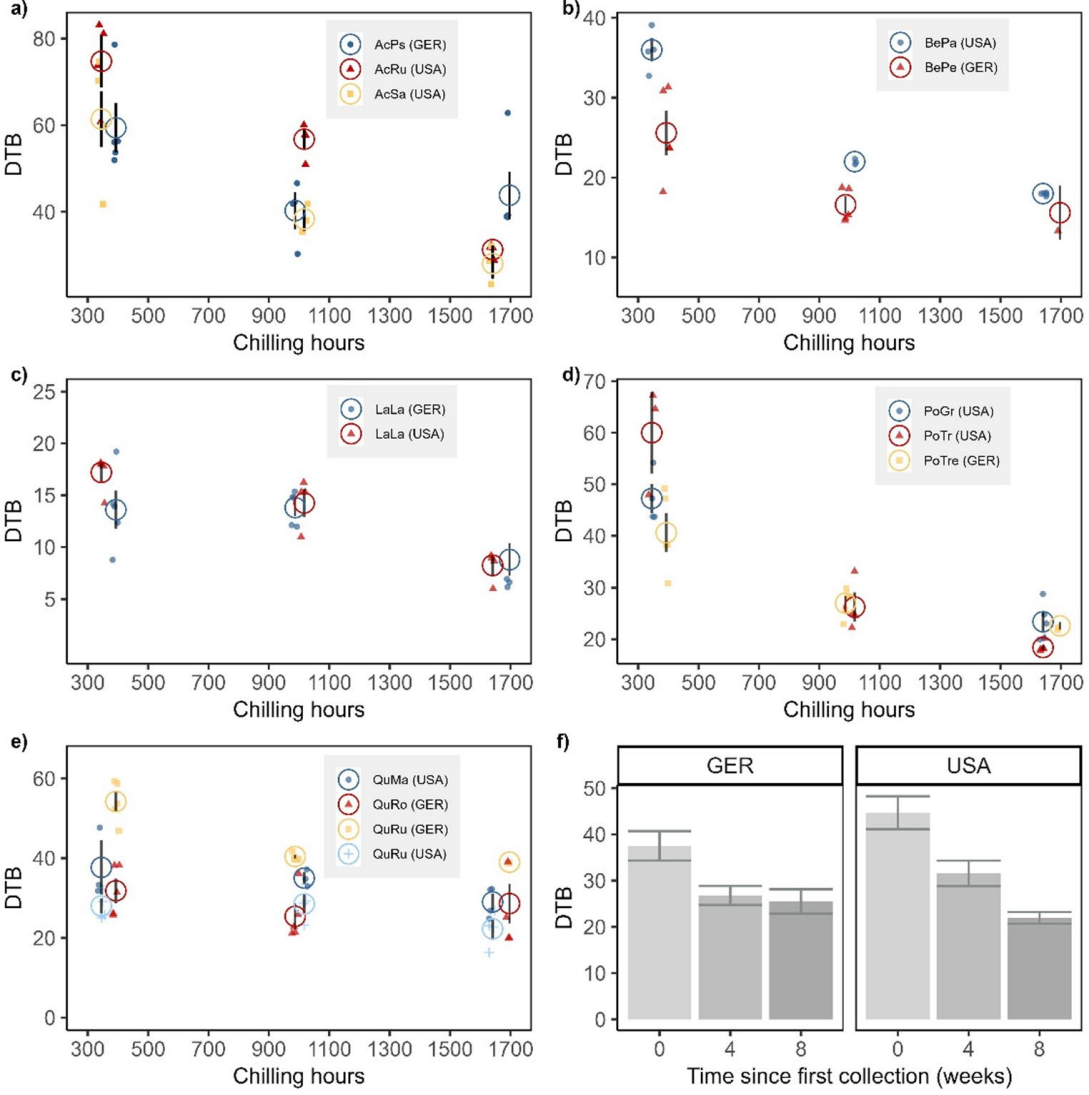
Effect of ambient chilling on the time to budbreak in twigs from boreal and temperate tree species in the USA and Germany. Twigs from Minnesota and Bavaria were collected during 3 different times throughout the winter of 2015/2016 and placed in growth chambers at 21°C/16°C, 16 hours photoperiod. The x axis shows accumulated chilling hours in both locations for all collections, i.e. hours below the chilling threshold of 5 °C since November 01, 2015. The panels (**a**-**e**) compare the days to leaf budbreak of species from both countries with the same genus. The circles show means of raw data (in color) and standard error bars (in black). Panel f shows the days to budbreak for all species and all collections (Germany and USA) with standard error bars

For both locations, DTB was significantly affected by the length of chilling exposure (USA: adj. R^2^ = 0.91; Germany: adj. R^2^ = 0.83, Fig. 4). Longer exposure to chilling reduced the time required to break bud (USA: *F*_1,75_=317.67, *P* < 0.001; Germany: *F*_1,80_=48.17, *P* < 0.001), and there was a significant difference between species (USA: *F*_7,75_=92.45, *P* < 0.001; Germany: *F*_5,80_=73.84, *P* < 0.001), but the interaction among species and chilling time was only statistically significant for the USA (USA: *F*_7,75_=7.32, *P* < 0.001). As an example, *Quercus rubra* buds from the first collection broke on average around 15 and 6 days later than buds from the third collection in Germany and the USA respectively. Because all species were able to break buds after the first collection, it can be assumed that chilling requirements for dormancy release were already fulfilled. At temperatures below freezing, there was still a continuous decrease in the need for forcing with increased chilling, suggesting that those temperatures were contributing to chilling accumulation. This is especially relevant for twigs from Minnesota, which were exposed to average daily temperatures of < 0 °C for the last 30 days of the experiment.

The timing to bud beak was significantly different for all species in Germany and the USA. At both locations, species of the *Acer* family had the slowest budbreak and required on average more chilling than all other species, while *Larix laricina* broke bud fastest and required on average less chilling (Fig. 4). However, because of steep declines in need for chilling in some species, these differences between DTB became less pronounced after the third collection when more chilling had been accumulated. For example, while after the first collection the difference between average DTB for *A. rubrum* and *L. laricina* was 43.5 days (74.75 (± 4.97 SE) for *A. rubrum* vs. 31.25 (± 0.75 SE) for *L. laricina*), after the third collection it was only 19.8 days (± 1.58 SE) for *A. rubrum* vs. 8.25 (± 0.75 SE) for *L. laricina*).

Budbreak percentage or survival was generally high for all species (> 50%), but more consistently close to 100% in Germany, except for *Q. rubra* from the third collection (see Figure S2c, d available as Supplementary Data). A comparison between the twigs from Experiment 1 that were artificially chilled for 4 weeks and twigs from Experiment 2 that were immediately forced after collection on Nov 21/27, 2015 (U.S/Germany) shows that all species were able to break bud, and that sufficient chilling had accumulated to break dormancy (see Figure S4 available as Supplementary Data).

## Discussion

The timing of spring budbreak can be a very important factor in the fitness of temperate and boreal tree species, partially because earlier springs can either allow for an earlier achievement of minimum resources that are needed to initiate reproduction (Journé et al. 2021) or trigger the risk of damage caused by spring frosts (Zohner et al. 2020), which can result in reduced productivity (Wang et al. 2025). Chilling as a phenological cue can impact budbreak timing, which can lead to ecological advantages or disadvantages. For example, if a species can benefit from warmer future winter temperatures more than others, it might be able to break bud and photosynthesize earlier, potentially outcompeting other species (see for example Uphus et al. 2021). Understanding the response of species to chilling is therefore important when making future predictions using phenological models. In our experiments, we exposed temperate and boreal species to different chilling lengths and temperatures, and we collected twigs during different times throughout the winter, after which we forced them in heated chambers, to elucidate the effect of chilling on their timing of leaf budbreak. Most species in both locations broke bud earlier after chilling with mild temperatures in comparison to colder temperatures, but even the twigs from our coldest treatments (−6.5 °C USA/−7 °C Germany) accumulated chilling and broke bud earlier after longer exposure to chilling (8 vs. 4 weeks). Additionally, boreal species in general broke bud faster in both locations than temperate species, and overall, species from Germany required less chilling than species from the USA.

### Timing of bud burst related to chilling temperature

Both our experiments confirm that chilling is an important aspect in the timing of spring leaf budbreak, which has been shown in many previous studies (see for example Gao et al. 2024). We show that for around half of the species in our experiment, exposure to various chilling temperatures lead to statistically significant differences in budbreak timing. However, chilling temperatures were not equally effective. While for most species, experimental chilling at around 4 °C decreased the need for forcing the most, this relationship was not always linear. For example, for *Q. macrocarpa*, after eight weeks, chilling was least effective in the 4.5 °C treatment, and for *A. saccharinum*, colder chilling increased the time to budbreak, which is in line with Wang et al. (2022) who found a negative correlation of warmer chilling and chilling accumulation. However, all our other species that were significantly affected by chilling temperature broke bud faster at warmer experimental chilling temperatures, indicating that, depending on location and current winter temperatures, climate change induced increases in mid-winter temperatures could potentially accelerate their budbreak in the future, which confirms findings from Harrington and Gould (2015) and Man et al. (2017). Our results suggest that overly simple generalizations regarding chilling requirements must be avoided and that species-specific chilling requirements need to be considered to better inform predictive models. It also suggests that the recent slowing trend of earlier spring phenology cannot simply be explained with warmer winter chilling for all species, and that whether warmer winter temperatures play a role depends on the region and the prevailing winter temperatures. This is also in agreement with Ettinger et al. (2020) who established that reduced chilling could only be detected in very warm regions. While we did not test for the effectiveness of chilling temperatures above 5 °C in experiment 1, it is possible that chilling is accumulated at temperatures up to 10 °C (Baumgarten et al. 2021). It would therefore be beneficial to include chilling temperatures between 5 °C and 10 °C in future experiments.

Recent studies found that the maximum and minimum temperatures during the day (Bigler and Vitasse 2019) and the diurnal temperature range between maximum and minimum temperatures (Huang et al. 2020) can have an important effect on the timing of budbreak (Meng et al. 2019). In this study we did not account for that, and twigs were chilled at constant temperatures in the dark, which also resulted in a rapid transition to 16 h of photoperiod in the growth chambers. Future experiments could incorporate even larger chilling ranges and differentiate between the diurnal temperature ranges.

Our results also indicate that chilling can accumulate below freezing temperatures, because very cold chilling (both ambient chilling and the artificial − 6.5/−7 °C treatments) contributed to advances in budbreak after increased chilling time, showing that buds accumulated chilling units at those low temperatures. This is in line with findings from Baumgarten et al. (2021). Contrary to those findings, for most of the species in our experiment, chilling above freezing was more effective and in both experiments, the survival of twigs at temperatures far below freezing was very high. In many phenological studies and models, a lower chilling threshold of 0 °C is used, and for boreal species, the lower threshold is sometimes set to −3.4 °C (Man et al. 2017). Our findings suggest that these thresholds should not be universally applied, and that all temperatures below freezing should be considered. It also supports the findings of Zohner et al. (2020), who found that in Europe, phenological models for boreal species performed better when below freezing temperatures were included, compared to models with a chilling range of 0–5 °C. However, we did not explore the effect of chilling just below freezing, which could be included in future chilling experiments.

Finally, our study clearly confirmed that across both countries, the length of chilling is an important factor in reducing the time to budbreak for 78% of the species in experiment 1 (*artificial chilling in chilling chambers)* and for 100% of the species in experiment 2 (*chilling at ambient temperature*). Generally, the effect of chilling time was stronger for species from the USA (Fig. 2a and b). Chilling time also had a stronger effect than chilling temperature (see also Baumgarten et al. 2021). For the three species that showed no response to chilling length in the chilling chamber experiment (*P. grandidentata*, *Q. robur*, *B. pendula*), it is possible that optimal chilling requirements were met after four weeks, and that consequently increased chilling had no added effect (Harrington et al. 2010).

### Species differentiation

The magnitude of phenological trends in our experiments differed among species in both countries. While some species required large amounts of chilling to reduce the need for forcing, others broke bud quickly after short exposure to chilling. For example, our results confirm findings from previous studies that *A. rubrum* requires high, while *L. laricina* requires low amounts of chilling (Harrington and Gould 2015; Nanninga et al. 2017). Two species, *P. grandidentata* and *Q. robur*, responded to neither chilling temperature nor length, and could therefore be classified as spring-only responders (Cook et al. 2012), or they could have very low chilling requirements. *P. grandidentata* did, however, show decreases in the time to budbreak with longer chilling under ambient chilling conditions. In previous studies, *Q. robur* has shown to have shallow dormancy with low chilling requirements (Baumgarten et al. 2021; Dantec et al. 2014; Wenden et al. 2020), which is in line with our findings. However, Laube et al. (2014) found *Q. robur* to be chilling sensitive, which contradicts our findings. The reasons for this discrepancy are unknown. They could be an artifact of the provenance of the twigs collected in Freising, differences in experimental setups, or the December temperatures in 2015 that were around 5 °C higher on average compared to 2011. In general, while future warmer winters might not strongly affect the budbreak of species with low chilling requirements, species with high chilling requirements might experience insufficient chilling. The consequence could be delayed budbreak (Man et al. 2021b) or erratic formation of leaves (Chuine et al. 2016; Harrington and Gould 2015; Man et al. 2017). Montgomery et al. (2020) found a stronger phenological response of boreal compared to temperate species in warmer plots in a warming experiment in Minnesota. We also showed that, apart from *P. tremula* in Germany, boreal species broke bud on average faster than temperate species, i.e., they required less chilling or possibly less forcing. Additionally, there was a trend towards faster budbreak with warmer chilling among boreal species. As a group, boreal species might therefore respond to warmer winters in northern latitudes in the future with earlier budbreak and earlier access to resources compared to temperate species, which could have consequences on ecological interactions and range limits. On the other hand, if they break bud too early, they might also be at higher risk of late frost damage (Man et al. 2017; Menzel et al. 2015). However, remote sensing has shown that between 1984 and 2013, North American boreal forests show more heterogeneous phenological changes with climate change than temperate forests (Melaas et al. 2018), emphasizing again the importance of understanding each species’ chilling and forcing preferences separately to make accurate predictions.

It is difficult to predict if the differences in response to chilling between species from Germany and the USA in this study are due to evolutionary factors or to responses to different environments or a combination of both. Provenance and common garden trials have shown that species or ecotypes from warmer areas (lower latitudes and altitudes) with early warm springs often have high chilling requirements that delay budbreak and protect from late spring frosts (Jensen and Hansen 2008; Salk 2020; Usmani et al. 2020). However, without the use of common garden trials, it is difficult to distinguish between the underlying mechanisms regarding chilling differences of species from different regions. Recent studies have shown that warmer winters, and hence warmer chilling temperatures, are currently advancing budbreak in cold regions, while they are delaying budbreak in warm regions (Vitasse and Rebetez 2018; Wenden et al. 2020; Yang et al. 2020). Further, Zohner et al. 2017 found higher chilling requirements in species from North America compared to Europe, and suggested the reason was a protective mechanism from more unpredictable temperature patterns in North America. Our study supports this finding. Comparing the same species or species with the same genus across both countries, we found that individuals grown in Germany generally broke bud faster than those from the USA, suggesting that they had lower chilling requirements or possibly lower forcing needs. This was most pronounced after 4 weeks, compared to 8 weeks of chilling (Fig. 2c) in experiment 1 (*artificial chilling in chilling chambers)* and for the first 2 collections (Fig. 4a) in experiment 2 (*chilling at ambient temperature*). However, this was not universal as the opposite was true for *Q. rubra*, both in experiment 1 (Fig. 3) and experiment 2 (Fig. 4). Despite some differences, the overall patterns of budbreak were very similar for species of the same family, which is especially apparent in the similar or even overlapping slopes of reduced DTB with increased chilling in experiment 2 (Fig. 4). This suggests that generalizations regarding the phenology of the same or similar species across different regions should be avoided.

When comparing the phenology of different species and/or ecotypes, we are aware that chilling is not the only important cue that could impact observations. It is hence possible that, additionally to different chilling preferences, boreal species and species from Germany also responded more readily to forcing accumulation in the growth chambers, which could have contributed to the faster budbreak. This study concentrates on and discusses the effect of chilling. The reduced need for forcing with increased length of chilling shows that chilling influenced budbreak timing, and how much the different forcing requirements impacted DTB is beyond the scope of this study.

### Implications for modeling phenology

Some recent studies discuss the relevance of chilling in budbreak and the necessity to better understand chilling to improve the predictability of phenological models (Ettinger et al. 2020; Flynn and Wolkovich 2018; Hänninen et al. 2019; Zohner et al. 2017). These models are relevant as they help in predicting future range limits and carbon sequestration, and they can support management decisions and the prioritization of management areas (Ettinger et al. 2020). After decades of observed advances in budbreak, a slowing down of this trend has been observed in recent years. Some studies relate this slow-down to reduced winter chilling (e.g. Fu et al. 2015b), or suggest it could be a statistical artifact (Ettinger et al. 2020). Others found that it could be caused by an earlier pre-season with warmer temperatures (Güsewell et al. 2017), reducing the amount of photoperiod during the forcing period (Fu et al. 2019b; Wenden et al. 2020). Our findings confirm that winter chilling is highly relevant in determining the timing of budbreak and should be considered as a factor in phenological models. However, in our experiment, warmer chilling temperatures were more effective at reducing DTB for many of the species, suggesting that the slowing trend of advanced DTB might not be related to chilling for all species. This again highlights the importance of understanding the species- and ecotype-specific responses to chilling, especially temperature ranges suitable for chilling accumulation. The effectiveness of chilling, which weighs the effect of chilling temperature on the need for thermal time, is commonly depicted as a bell-shaped curve with a peak of effectiveness calculated as one chilling unit, and a decrease in effectiveness up to an upper and lower threshold (Chuine 2000; Sarvas 1974). The species in our experiment do not show this pattern, with the exceptions of *L. laricina* (USA), and *Q. robur* after four weeks of chilling. It is possible that the peaks of species were located outside of the chilling range used in this experiment. We also set the upper temperature threshold in the chilling chambers to 5 °C (Richardson et al. 1974), the estimated lower threshold for forcing, because at higher temperatures it would have been impossible to distinguish the effect of chilling and forcing (Hänninen 2016). We did, however, use a large range of chilling temperatures of 11 °C in both countries, which suggests that maybe the bell-shaped curve is not the ideal way of calculating chilling accumulation for all the species and that chilling can be accumulated at large temperature ranges. It is possible that for those species with no significant response to chilling temperature, simpler threshold models are more suitable (Hänninen 2016). For the two species (*P. grandidentata*, *Q. robur*) that showed neither sensitivity to chilling time nor to chilling temperature, it might be possible to exclude chilling as a parameter from phenological models.

## Conclusions

Our experiments confirmed that chilling is an important cue for the spring budbreak in temperate and boreal tree species in both the USA and Germany. In contradiction to current models, our data clearly shows that below freezing temperatures contribute to chilling accumulation and therefore these need to be incorporated into phenological models. For most species in our experiment, increased chilling duration reduced the DTB, and half of the species experienced changes in the DTB when chilled at different temperatures. Because the magnitude and direction of change with changing chilling temperatures were not linear, we expect varying responses of species and ecotypes to warming winter temperatures. Generally, boreal species broke bud earlier than temperate species, and patterns of budbreak were similar. Our results highlight the need for further experiments to best understand species-specific chilling requirements, both regarding temperature and time, and that generalizations should be avoided, especially in phenological models.

## Supplementary Information

Below is the link to the electronic supplementary material.

Supplementary Material 1

## Data Availability

The datasets generated during and/or analysed during the current study and the code used to analyze the data are available in the University of Minnesota repository, https://hdl.handle.net/11299/275429.

## Footnotes

1 Data from 01/01–01/04/2010 missing.
