# Supplementary material for: Comparison of chilling requirements of boreal and temperate tree species in Germany and North America
Source: Int J Biometeorol. 2026 Jan 30;70(2):43. doi: 10.1007/s00484-026-03129-0 (PMC12858481; doi:10.1007/s00484-026-03129-0)
Supplement: Supplementary file 1 — Supplementary Material 1 [file 484_2026_3129_MOESM1_ESM.pdf]

Comparison of chilling requirements of boreal and temperate tree species in Germany and North America

Claudia Nanninga*,**, Rebecca A. Montgomery*, Annette Menzel***, Julia Laube****

**Department of Forest Resources, University of Minnesota, 1530 Cleveland Ave N, St. Paul, MN 55108, U.S.A.*

***Email: nanni003@umn.edu, Phone: 612 987 8450, ORCID: 0000-0003-2361-6839*

**** TUM School of Life Sciences, Department of Ecoclimatology, Technical University of Munich, Hans-Carl-von-Carlowitz Platz 2, 85354 Freising, Germany*

*****University of Applied Sciences, Department of Engineering Ecology and Landscape Developent, Am Hofgarten 6, 85354 Freising, Germany*

Journal: International Journal of Biometeorology

Supplemental Information (SI): Appendix.

Appendix Figures:

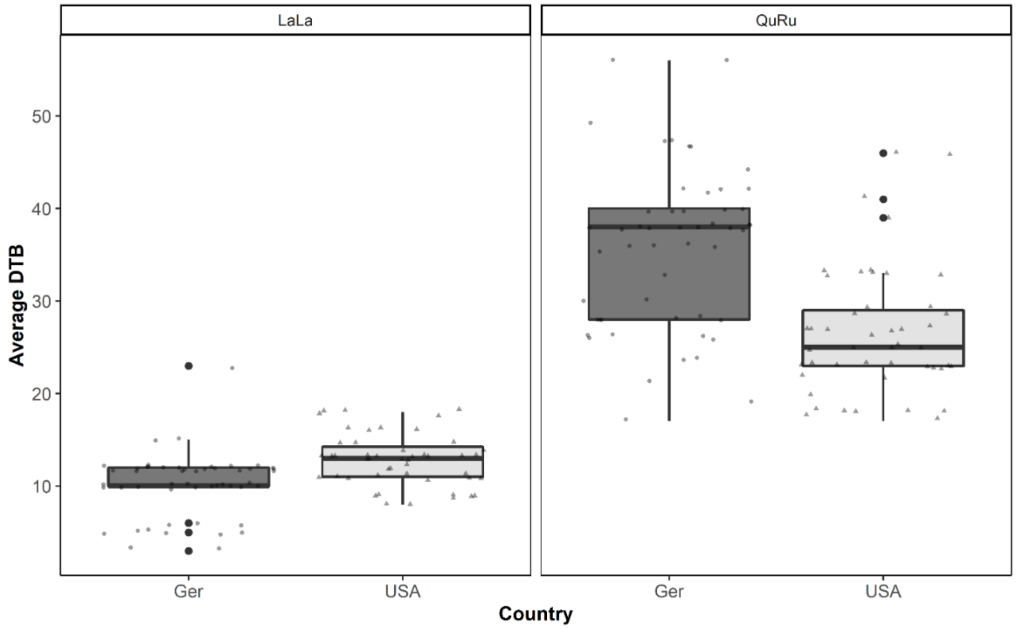

Figure S1: Effect of experimental chilling on the time to bud break in twigs from *Larix laricina* and *Quercus rubra* in the USA and Germany. Twigs were chilled at 3 different temperatures and after 4 and 8 weeks exposed to 21°C/16°C in growth chambers until leaf buds burst. The graph depicts the days to leaf bud break across all temperature and length treatments for both species and both countries.

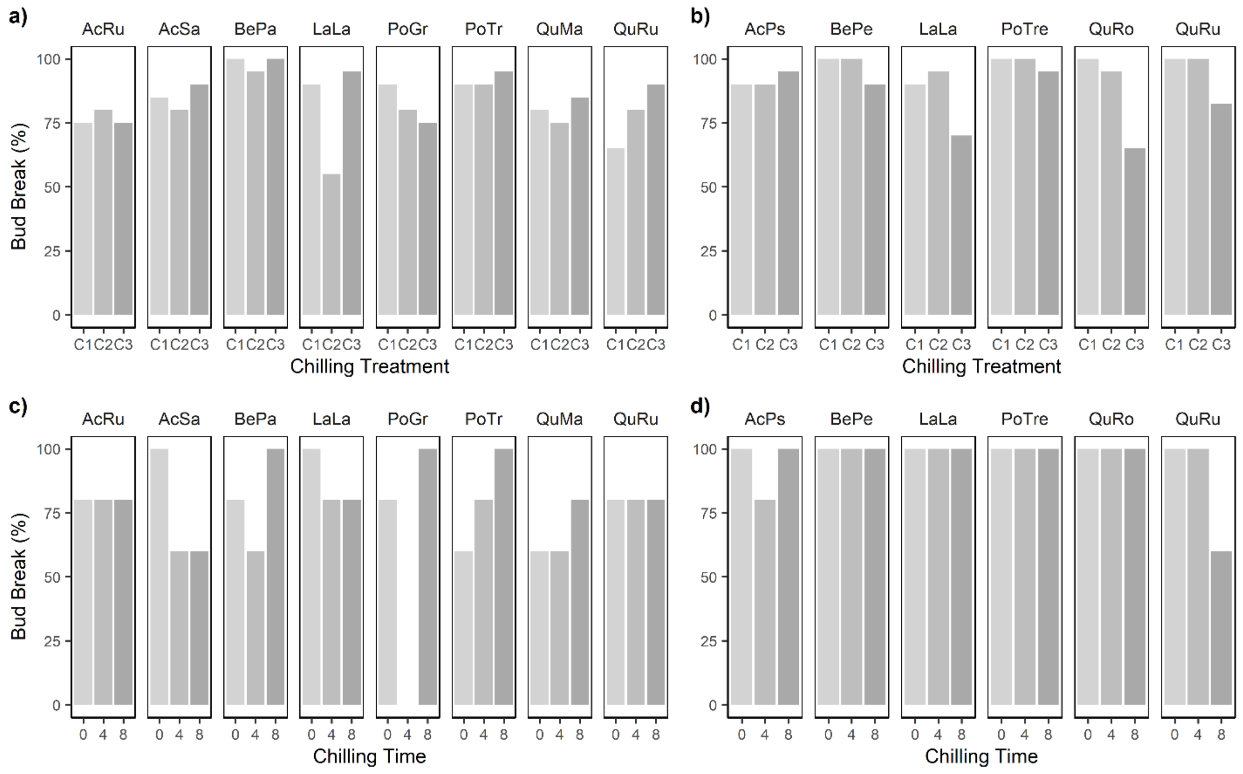

Figure S2: The graph shows the percentage of twigs with at least one broken bud experiment 1: artificial chilling in chilling chambers (a) USA, b) Germany (C1=coldest chilling treatment, C2=mid chilling treatment, C3=warmest chilling treatment); and from experiment 2: chilling at ambient temperature (c) USA, d) Germany (0, 4, and 8 weeks of ambient chilling time after the first collection). Data for P. grandidentata after 4 weeks of ambient chilling is missing.

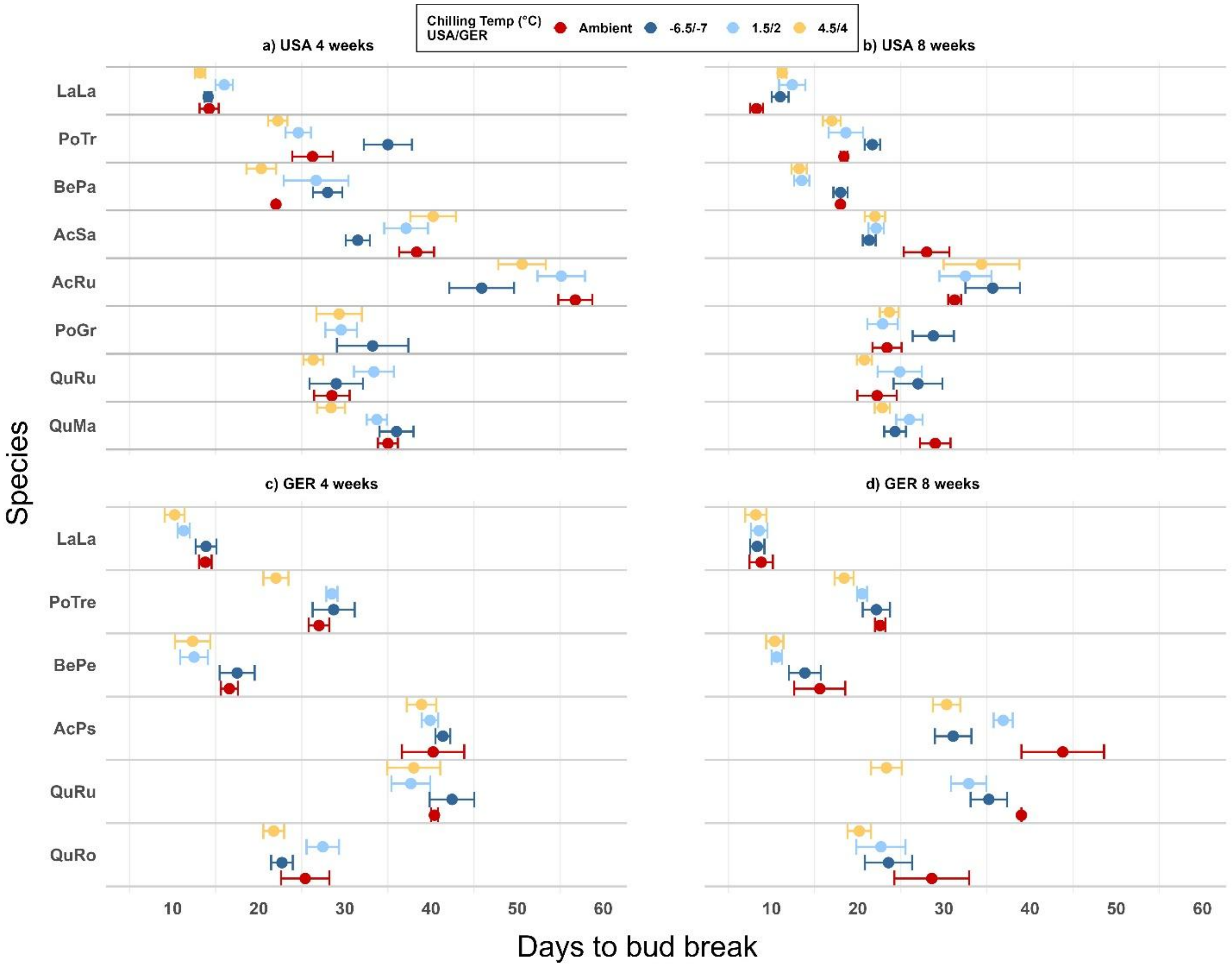

Figure S3: Effect of experimental chilling on the time to bud break in twigs from 8 species in the USA after 4 weeks (a) and 8 weeks (b) of artificial chilling and 6 species in Germany after 4 weeks (c) and 8 weeks (d) of artificial chilling (C1, C2, C3). The individual twigs from the control group (red) were forced after 4 weeks of ambient chilling (a and c) and 8 weeks of ambient chilling (b and d). The dots show the mean of the raw data, error bars show the 95% Confidence Intervals. Data for *P. grandidentata* at ambient temperatures after 4 weeks is missing.

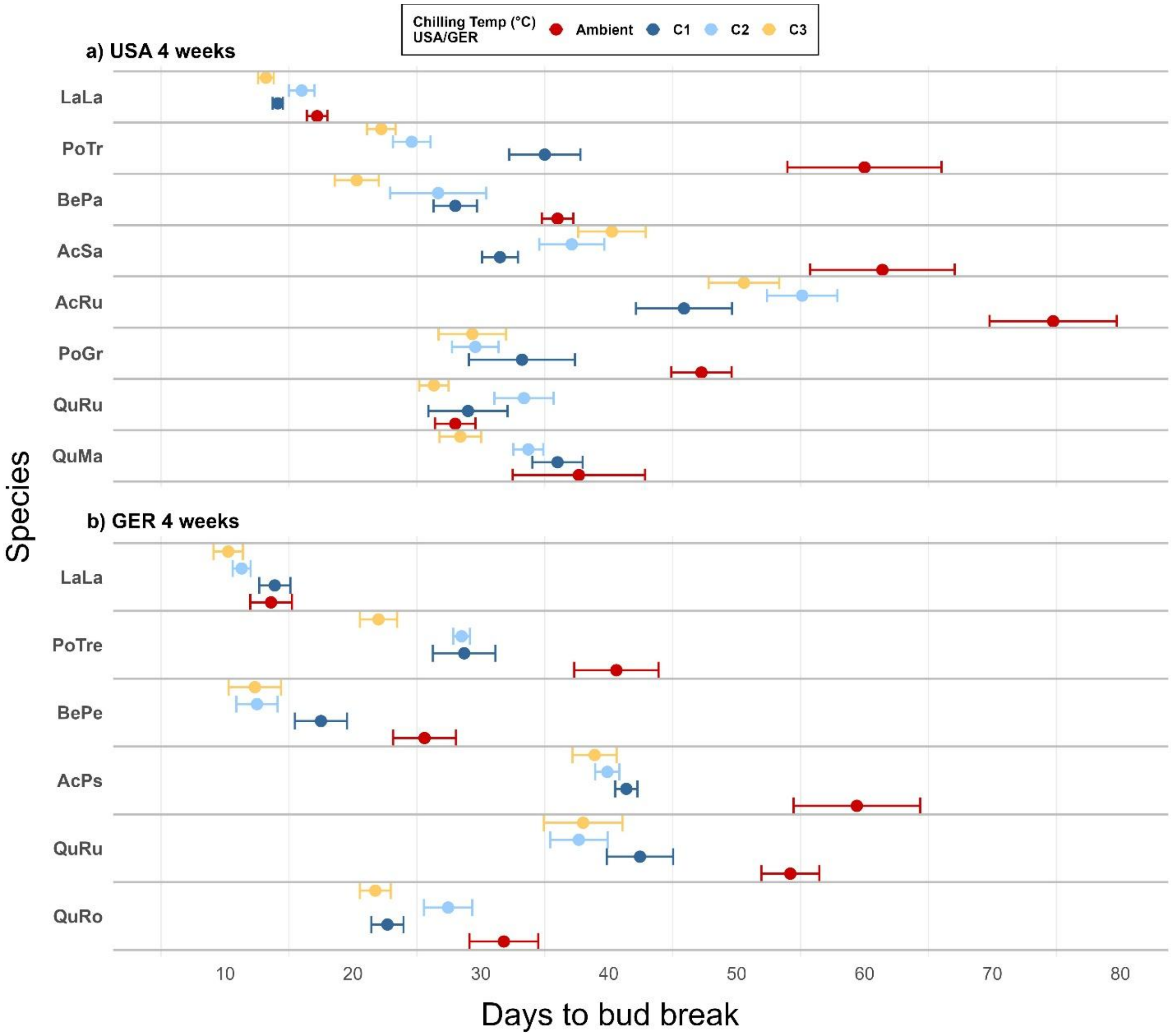

Figure S4: Effect of experimental chilling on the time to bud break in twigs from 8 species in the USA (a) and 6 species in Germany (b) after 4 weeks of artificial chilling (C1, C2, C3). The individual twigs from the control group (red) were forced right after the collection on November 21 (U.S.) and 27 (Germany) of 2015, and they were not exposed to artificial chilling. The dots show the mean of the raw data, error bars show se.
